# Monitoring *Plasmodium vivax* chloroquine sensitivity along China-Myanmar border of Yunnan Province, China during 2008–2013

**DOI:** 10.1186/1475-2875-13-364

**Published:** 2014-09-15

**Authors:** Hui Liu, Heng-lin Yang, Lin-hua Tang, Xing-liang Li, Fang Huang, Jia-zhi Wang, Chun-fu Li, Heng-ye Wang, Ren-hua Nie, Xiang-rui Guo, Ying-xue Lin, Mei Li, Jian-Wei Xu

**Affiliations:** Yunnan Institute of Parasitic Diseases, Puer, 665000 China; National Institute of Parasitic Diseases, Chinese Center for Disease Control and Prevention, Shanghai, 200025 China; Tengchong County Center for Disease Control and Prevention, Tengchong, China; Yangjiang County Center for Disease Control and Prevention, Tengchong, China

**Keywords:** *Plasmodium vivax*, Chloroquine, Resistance, China-Myanmar border

## Abstract

**Background:**

*Plasmodium vivax* is the most widespread of the malaria parasites infecting human hosts. In malaria-eliminating settings, both imported and local malaria predominantly occurs in border areas, and most of them are *P. vivax*. Chloroquine (CQ) is the first-line drug for *P. vivax* treatment in China. To understand CQ sensitivity in *P. vivax*, *in vivo* monitoring of CQ resistance was conducted along the China-Myanmar border from 2008 to 2013.

**Methods:**

Eligible patients with mono-infections of *P. vivax* were recruited to this study after obtaining full informed consent. CQ tablets for different categories of kg body weight ranges were given once a day for three days. Patients were followed up for 28 days. PCR was conducted to distinguish between re-infection and recrudescence, to confirm the *Plasmodium* species. The data were entered and analysed by the Kaplan-Meier method. Treatment outcome and sensitivity were classified according to the WHO recommended standards.

**Results:**

603 patients were completed valid follow-up. The fever clearance time and asexual parasite clearance times were, respectively, 22.2 ± 10.2 and 38.1 ± 12.6 hours. 594 (98.5%) patients were adequate clinical and parasitological response (ACPR), and nine (1.5%) patients, who were late clinical failure (LCF) or resistant response level I (RI), were imported from the neighbouring districts of Myanmar.

**Conclusion:**

In terms of efficacy, CQ is still effective for vivax malaria treatment. *Plasmodium vivax* CQ sensitivity had not significantly changed along the China-Myanmar border of Yunnan Province, China.

## Background

Malaria is still a global public health problem. The World Health Organization (WHO) estimated that between 2000 and 2010, global malaria incidence decreased by 17% and malaria-specific mortality rates decreased by 26%. Reported malaria cases have reduced by more than 50% in 34 of the 99 malaria-endemic countries [1]. China has declared a national policy for malaria elimination by 2020 [2, 3]. In eliminating settings, malaria predominantly occurs in border areas, and imported cases tend to make up the majority of recorded cases caused by *Plasmodium vivax* [4]. Resistance to anti-malarial drugs has often threatened malaria elimination efforts and historically has led to the short-term resurgence of malaria incidence and deaths [5]. This can jeopardize progress and investment in combating malaria [6]. *Plasmodium vivax* is the most widespread of the malaria parasites infecting human hosts. Although there were an estimated 72–80 million *P. vivax* infections each year, it has not received as much public and scientific attention as *Plasmodium falciparum* [7]. In fact*, P. vivax* can lead to a disabling disease that can be fatal, and exacts a similar economic burden as falciparum malaria [8]. Finding and treating the *P. vivax* infection to prevent onward transmission is the determinant intervention. Chloroquine (CQ)-resistant *P. vivax* was first described in 1989 in Papua New Guinea [9], and the decline in the efficacy of CQ has been reported in some geographical sites [10]. CQ is the first-line drug for *P. vivax* treatment in China. Understanding *P. vivax* sensitivity to CQ is the basis of vivax malaria treatment with CQ. Knowledge of drug sensitivity is also needed to guide the local drug policy, so WHO strongly encourages countries in which *P. vivax* is endemic to carry out pilot studies to monitor the efficacy of CQ [11]. From 2008 to 2013, *in vivo* monitoring of CQ resistance in *P. vivax* was conducted to determine the dynamics of *P. vivax* sensitivity to CQ in the field along the China-Myanmar border.

## Methods

### Surveillance sites and time

**Figure 1:**
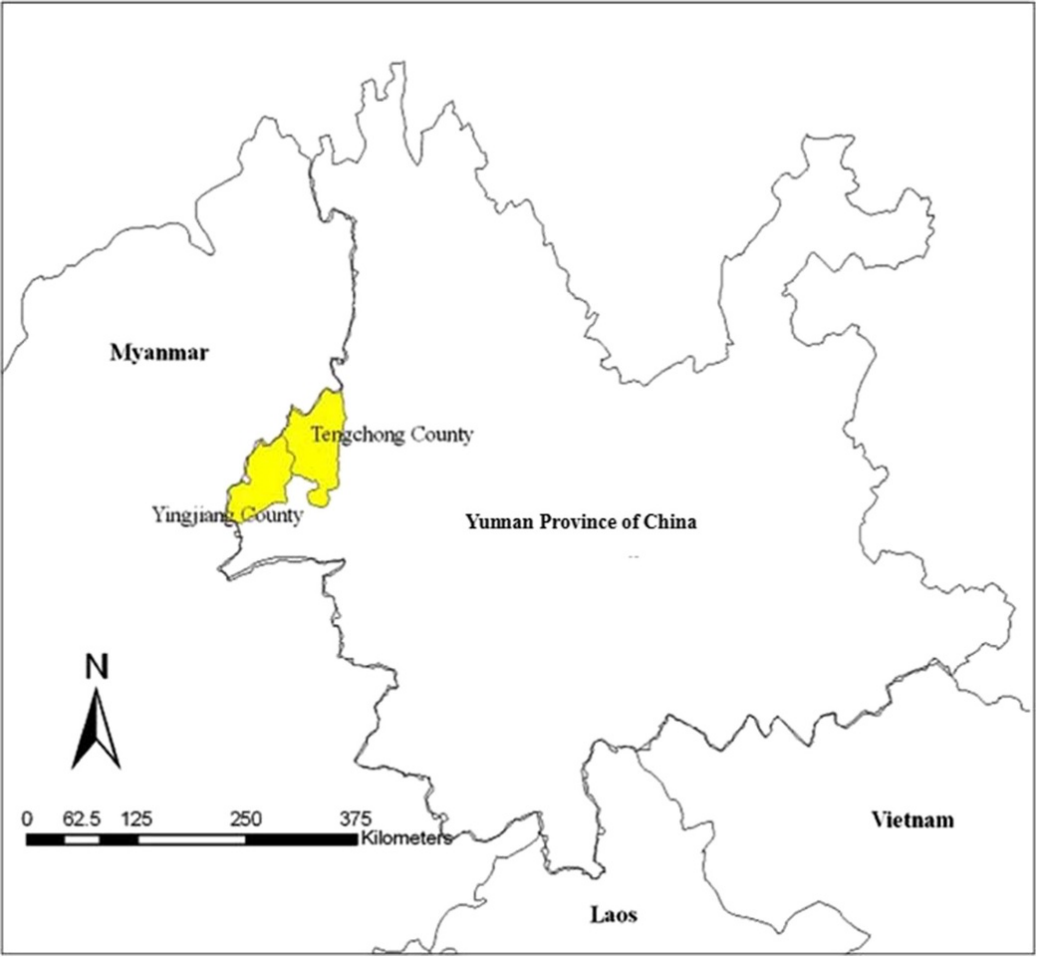
**The location of Tengchong and Yingjiang counties relative to neighbouring countries.**

Surveillance was carried out in Tengchong and Yangjiang, two counties on the China-Myanmar border (Figure 1). One of the main reasons for this selection of surveillance sites was that suitable vivax malaria patients were difficult to find in other parts of China. Most of the malaria cases in the two counties were imported from neighbouring districts of Myanmar. The surveillance activities were conducted in Tengchong County in 2008 and 2009, and expanded to Yingjiang County in 2010 and 2011, and only carried out in Yingjiang County because of so few malaria cases in Tengchong in 2012 and 2013.

### Patients and recruiting criteria

Patients whose axillary temperature was ≥37.5°C or history of fever during the previous 24 hours were diagnosed based on microscopy of thick and thin blood smears. Patients with mono-infections of *P. vivax* were recruited to this study after obtaining full informed consent. Only patients older than one year presenting with parasite density 500–120,000 parasites per μL were enrolled into the study. Imported malaria was identified as patients who had traveled from endemic areas of Myanmar within one month and were diagnosed as malaria in China [3]. Patients were excluded from the study if any of the following criteria were present: (1) positive pregnancy test or breastfeeding; (2) complicated malaria; (3) having taken any anti-malarial, sulpha, tetracycline and sulphone drugs within the previous seven days; (4) history of hypersensitivity to any of the study drugs; (5) severe dysfunction with kidney, liver and heart; (6) over 60 years old; and, (7) unable to follow up.

### Drug and administration

CQ (GYZZ H31020423, 155 mg base per tablet) was made by Shanghai Sino-West Pharmaceutical Corp, China, and provided by The West Pacific Office of World Health Organization (WPRO/WHO). CQ was given once a day for three days. The doses were recommended by WPRO/WHO too. For convenient administration of the drugs, the doses were calculated into tablets for different categories of kg body weight ranges (Table 1) [12]. Based on the recommendation of the China Ministry of Health, a standard adult (≥16 years) dosage of primaquine tablets (22.5 mg/day) was given for treatment of liver-stage parasites over eight days after completion of 28-day follow-up. The age based dosing was used for children, 3.75-5.625 mg/day for age 1–3 years, 7.5 mg/day for age 4–6 years, 11.25 mg/day for age 7–12 years and 16.875 mg/day for age 13–15 years over eight days [12].

**Table 1:** **Number of chloroquine tablets (155 mg base per tablet) for each category of body weight range**

Body weight range (kg)	Number of tablets	Number of tablets	Number of tablets	Total tablets
	Day 1	Day 2	Day 3	
≤ 12	1	0.5	0.5	2
13-24	2	1	1	4
24.1- 36	3	1.5	1.5	6
36.1- 48	4	2	2	8
>48	5	2.5	2.5	10

### Laboratory and *in vivo*clinical monitoring

Parasite microscopy was conducted on admission and every 12 hours while the patients stayed in hospital over the following seven days. Then patients were discharged from hospital and further parasitological examinations were performed on days 14, 21 and 28. Malaria blood films were stained with Giemsa and parasites were counted per 500 white blood cells. The number of parasites was calculated as per ul of blood by the level of 8,000 of leukocyte per ul [13]. A filter-paper dried blood spot (about 100 ul blood) was prepared on admission, days 3, 7, 14, 21, and 28 for polymerase chain reaction (PCR) to distinguish between re-infection and recrudescence, to confirm the *Plasmodium* species or to detect mixed infection. Improved Chelex 100 ion exchange method was used to extract DNA from blood filter paper samples, nested PCR and allele specific PCR techniques, agarose gel electrophoresis analysis and dot/southern blotting probe hybridization were employed for amplification, resolution and identification of the circumsporozoite protein (CSP) gene to distinguish reinfection from recrudescence or relapse. The primary PCR primers were 5′- TCCCCACGCACTGCAAAC ACAAT-3′ (F) and 5′- CCGCAGGAGGTGCCACGTATAATT-3′(R). The secondary PCR primers were 5′- GAAAATAAGCTGAAACAAC-3′ (F) and 5′-TACGTCACATTGGAC ACCT-3′(R) [14]. In case of failure after day 7, patients whose PCR results were unknown would be excluded from the analysis in accordance with the standardized WHO protocol [11]. The treatment outcome was assessed on the basis of parasite clearance from the blood. Parasite clearance was defined as no parasite per 500 white blood cells by two continuous every 12-hour microscopy. The primary endpoint was the 28-day cure rate. Cure was defined as elimination of the symptoms and asexual blood stages of the malaria parasites that caused the patient to seek treatment. Fever clearance was defined as axillary temperatures < 37.1°C in duration of 24 hours [15].

### Classification standards for treatment outcome and sensitivity

Treatment outcome was categorized based on the WHO definitions for early treatment failure (ETF), late clinical failure (LCF), late parasitological failure (LPF), and adequate clinical and parasitological response (ACPR). The ETF definition was to conform to any one of the criteria: (1) danger signs or severe malaria on day 1, 2 or 3, in the presence of parasitaemia; (2) parasitaemia on day 2 higher than on day 0, irrespective of axillary temperature; (3) parasitaemia on day 3 with axillary temperature ≥37.5°C; and, (4) parasitaemia on day 3 ≥ 25% of count on day 0. The LCF definition was to satisfy any one of the criteria: (1) danger signs or severe malaria in the presence of parasitaemia on any day between day 4 and day 28 in patients who did not previously meet any of the criteria of ETF; and, (2) presence of parasitaemia on any day between day 4 and day 28 with axillary temperature ≥37.5°C in patients who did not previously meet any criteria of ETF. LPF definition was to satisfy presence of parasitaemia on any day between day 7 and day 28 with axillary temperature <37.5°C in patients who did not previously meet any of the criteria of ETF or LCF. ACPR definition was to satisfy absence of parasitaemia on day 28, irrespective of axillary temperature, in patients who did not previously meet any criteria of ETF, LCF or LPF [11].

Resistant responses (S, RI–RIII) were categorized according to the WHO criteria [13]. Susceptible parasites (S) were defined if asexual stage parasites had a ≥75% reduction within 48 hours after drug treatment and slides were negative for two consecutive days within seven days, and no recrudescence occurred within the 28-day follow-up period. RI definition was 75% reduction in parasitaemia within 48 hours after initiation of therapy and parasite clearance within seven days but recrudescence within 28 days; RII was ≥75% reduction of initial parasitaemia within 48 hours of drug treatment, but without parasite clearance within seven days; RIII was parasitaemia ≥25% of initial parasite count at 48 hours after initiation of therapy. Patients with resistant parasites (RI-RIII) were subsequently given a standard adult dosage of dihydroartemisinin-piperaquine tablets following the Ministry of Health recommended two-day treatment regimen (dihydroartemisinin 160 mg and piperaquine 1280 mg/day) [13].

### Statistical analysis

The data were entered by Microsoft Office Excel 2007 and analysed by the Kaplan-Meier method [11, 16]. The patients of loss to follow-up and withdrawal from the study were not involved into the analysis.

### Ethical approval

According to the Helsinki Declaration, ethical approval for the study was granted by the Ethics Committee of Yunnan Institute of Parasitic Diseases, China. The purpose of the study was explained and then approval was sought from patients and their caretakers. Informed written consent was obtained from patients or from carers of child patients. All results were kept confidential and were unlinked to any identifying information.

## Results

During 2008–2013, a total of 750 vivax malaria patients were recruited in the study, 14 withdrew, 133 were lost at follow-up, and 603 completed valid follow-up, of which 531 (88.1%) were Burmese (Table 2). The fever clearance time (FCT) and asexual parasite clearance times (APCT) were, respectively, 22.2 ± 10.2 and 38.1 ± 12.6 hours. The results showed that 594 (98.5%) of the patients were ACPR, and nine (1.5%) LCF, without ETF and LPF. Two LCF patients were observed in 2010, one in 2012 and six in 2013 (Table 3). All nine LCF patients responded well to the two-day treatment regimen of dihydroartemisinin-piperaquine tablets. The result of parasite sensitivity classification was the same as the treatment outcome. The number of susceptible parasites was 594 and the RI was nine (Table 4). The PCR identified nine (1.5%) as recrudescence within day 28, one recrudescence on day 14 and 28, respectively, in 2010; one on day 28 in 2012; one on day 14 and 21, respectively, and four others on day 28 in 2013 (Tables 3 and 4). The PCR did not identify any new infection of *P. vivax.* All nine patients displaying RI and LCF were from the neighbouring districts of Myanmar and no significant differences were identified between different classifications. 1.5% (9/601) of LCF among imported cases from Myanmar including 531 Burmese and 70 Chinese who contracted malaria in Myanmar and come back to China for treatment versus 0% (0/2) locally acquired in China (x^2^ = 0.03, P = 0.862); 1.7% (9/531) of LFC in Burmese and 0% (0/72) in Chinese (x^2^ = 0.35, P = 0.552). 1.0% (3/287) of LFC in adults (≥16 years) versus 1.9% (6/316) in children (x^2^ = 0.28, P = 0.598).

**Table 2:** **Baseline characteristics of vivax malaria patients in Tengchong and Yingjiang counties, Yunnan, China**

	2008 (n = 22)	2009 (n = 9)	2010 (n = 84)	2011 (n = 43)	2012 (n = 123)	2013 (n = 322)	Total (n = 603)
**Sex**							
Male	20 (89.9%)	8 (88.9%)	63 (75.0%)	28 (65.1%)	69 (56.1%)	165 (51.2%)	353 (58.5%)
Female	2 (9.1%)	1 (11.1%)	21 (25.0%)	15 (34.9%)	54 (43.9%)	157 (48.8%)	250 (41.5%)
**Nationality**							
Chinese	22 (100%)	9 (100%)	18 (21.4%)	10 (23.3%)	8 (6.5%)	5 (1.6%)	72 (11.9%)
Burmese	0	0	66 (78.6%)	33 (76.7%)	115 (93.5%)	317 (98.4%)	531 (88.1%)
**Age (years)**							
Mean ± SD	20.2 ± 1.4	22.4 ± 4.8	28.4 ± 8.2	33.5 ± 6.4	26.2 ± 10.2	18.3 ± 9.6	25.2 ± 6.8
Range	23-50	31-53	2-59	2-60	2-59	1-60	1-60
**Body temperature (°C)**							
Mean ± SD	38.1 ± 0.4	38.1 ± 0.8	38.5 ± 0.5	39.3 ± 0.5	38.8 ± 0.6	38.2 ± 0.8	38.5 ± 0.7
Rang	36.3-39.5	37.0-39.4	36.8-40	37.1-41.0	36.6-40.0	36.5-40.8	
**Parasite count (per μl)**							
Geometric mean	7,621	6,327	8,675	6,732	8,976	8,156	7,881
Range (per μl)	556-25,606	1,582-18,334	521-102,500	663-99,130	546-111,960	586-97,846	521-111,960

**Table 3:** **Treatment responses of vivax malaria patients in Tengchong and Yingjiang counties, Yunnan, China**

	2008 (n = 22)	2009 (n = 9)	2010 (n = 84)	2011 (n = 43)	2012 (n = 123)	2013 (n = 322)	Total (n = 603)
ETF	0	0	0	0	0	0	0
LCF	0	0	2 (2.4%)	0	1 (0.8%)	6 (1.9%)	9 (1.5%)
LPF	0	0	0	0	0	0	0
ACPR	22 (100%)	9 (100%)	82 (97.6%)	43 (100%)	122 (99.2%)	316 (98.1%)	594 (98.5%)

Note: ETF = early treatment failure, LCF = late clinical failure, LPF = late parasitological failure, and ACPR = adequate clinical and parasitological response.

**Table 4:** **Clinical resistance of** ***Plasmodium vivax*** **in Tengchong and Yingjiang counties, Yunnan, China**

	2008 (n = 22)	2009 (n = 9)	2010 (n = 84)	2011 (n = 43)	2012 (n = 123)	2013 (n = 322)	Total (n = 603)
S	22 (100%)	9 (100%)	82 (97.6%)	43 (100%)	122 (99.2%)	316 (96.1%)	594 (98.5%)
R I	0	0	2 (2.4%)	0	1 (0.8%)	6 (3.9%)	9 (1.5%)
R II	0	0	0	0	0	0	0
R III	0	0	0	0	0	0	0

Note: S = susceptible, RI = resistant level I, RII = resistant level II, RIII = resistant level III.

## Discussion

WHO encourages countries in which *P. vivax* is endemic to carry out pilot studies to monitor CQ efficacy [11]. CQ is the first-line drug for treatment of blood stage infection of *P. vivax* and it has been used in treatment of vivax malaria for more than 50 years in China. The study monitored CQ therapeutic efficacy for uncomplicated vivax malaria on the China-Myanmar border from 2008 to 2013. The cumulative success rate for *P. vivax* treatment was 98.5%. All nine treatment failures were LCF or RI resistance. Wei *et al*. carried out a study on CQ therapeutic efficacy for *P. vivax* treatment in China-Vietnam border from 1989 to 1998. They found *P. vivax* resistance to CQ in 1989 and identified three RI, eleven RII and three RIII in the ten years. The cumulative failure rate (CFR) was 37.1% (17/47) [17]. Yang *et al*. observed 100 vivax malaria patients in central Yunnan Province in 1995, two RI and two RII were identified and the CFR was 4.0% (4/100) [18]. In the two studies, patients with RI-RII *P. vivax* were subsequently given a standard dosage of intramuscular artemether injections following the Ministry of Health’s recommended five-day treatment regimen (160 mg on day 0 and 80 mg daily for four days) [17, 18]. Liang *et al*. reported one RI and CFR 1.9% (1/54) on the same China-Myanmar border in 2008. They treated the CQ-resistant parasites with the two-day treatment regimen of dihydroartemisinin–piperaquine tablets [19]. Six CQ-resistant isolates were identified through *in vitro* microtest in central China in 2005 [20]. The *P. vivax* resistance to CQ was also found in Myanmar in 1990s [21–23]. Guthmann *et al*. reported that 34.1% (80/235) of vivax malaria patients with CQ treatment had recurrent parasitaemia and were considered treatment failures in southern Myanmar between December 2002 and April 2003 [24]. In other parts of the world, CQ resistance in *P. vivax* has been documented across the Indonesian archipelago [25], South Korea [26], Turkey [27], the Horn of Africa [28], and South America [29]. These studies showed that there are increasing worldwide reports of CQ resistance in *P. vivax*, however they did not indicate significant change of CQ resistance [9, 30].

The pace of CQ resistance development in *P. vivax* depends on numerous factors, such as drug selective pressure, endemicity, host immunity, and varies from region to region. CQ was used for clinical and prophylactic treatment of both *P. vivax* and *Plasmodium falciparum* in China in 1980s. The drug policy was changed in response to *P. falciparum* drug resistance, and then CQ was used only for vivax malaria treatment since 1990s. This may be one of reasons that the CFR (1.5%) of the study is much lower than the CFR (37.1%) between 1989 and 1998 in Yunnan Province, China [17, 18]. One of Wei *et al*. and Yang *et al*. studies’ technical limitations was not to use PCR to distinguish between re-infection and recrudescence to confirm the *Plasmodium* species or to detect mixed infection, and to exclude microscopic misidentification of *P falciparum* as *P vivax* when *P falciparum* prevalence was higher during 1989–98 and young ring stages difficult to distinguish. These might have increased their CFRs. Yunnan is a large province, with Wei *et al.* doing their study on the China-Vietnam border and Yang *et al.* in central Yunnan, whereas the current and other studies reported were done on the China-Myanmar border. Geographic differences may be one of explanations for reported differences too. Tengchong and Yingjiang are in a malaria-eliminating phase, so PCR did not identify any new infection within day 28. LCF of the study was observed in 2010, 2012 and 2013 and all nine LCF patients were imported from neighbouring areas of Myanmar. This shows that CQ resistance has not increased in recent years in Yunnan Province. CFR 1.9% (1/54) in 2008 [19], 2.4% (2/84) in 2010, 0.8% (1/123) in 2012 and 1.9 (6/322) in 2013 showed that CQ resistance did not significantly change (x^2^ = 0.86, P = 0.834) along the China-Myanmar border.

Despite PCR being used to distinguish between re-infection and recrudescence, to confirm the *Plasmodium* species or to detect mixed infection in the study, *P. vivax* can relapse from long-lasting liver stages [11, 23]. It is one limitation for the study and remains a challenge for monitoring *in vivo P. vivax* drug resistance. However, most of *P. vivax* in tropical areas is usually an early relapsing strain despite the late relapsing strain (temperate zone strains) exists in some high altitude (around 3,000 metres above the sea level) areas in Yunnan. The study was carried out in tropical areas and most of malaria patients were infected in Myanmar, so most of them should be tropical strain or early relapsing strain. This would reduce the PCR limitation in distinguishing recrudescence from relapse. Another limitation of the study is the change of surveillance sites, in Tengchong in 2008–2009, in both Tengchong and Yingjiang in 2010–2011, and in Yingjiang alone in 2012–2013. However, both monitoring sites are in the same section of the China-Myanmar border, so the study results indicate *P. vivax* sensitivity to CQ in the target region. The third limitation is that the number of valid study cases was only 22 in 2008, nine in 2009 and 43 in 2011, which may be the reason that CQ resistance was not found in those three years. The forth limitation is that children < 1 year and over 60 years old persons were excluded from the study, so the results may not be applicable to these two age groups.

## Conclusion

In terms of efficacy, CQ is still effective for vivax malaria treatment. *Plasmodium vivax* CQ sensitivity has not significantly changed on the China-Myanmar border of Yunnan Province, China.
